# The impact of voxelotor treatment on leg ulcers in patients with sickle cell disease

**DOI:** 10.1002/ajh.26101

**Published:** 2021-02-19

**Authors:** Caterina P. Minniti, Jennifer Knight‐Madden, Margaret Tonda, Sarah Gray, Joshua Lehrer‐Graiwer, Bart J. Biemond

**Affiliations:** ^1^ Department of Hematology Montefiore Medical Center/Albert Einstein College of Medicine Bronx New York; ^2^ Sickle Cell Unit, Caribbean Institute for Health Research University of the West Indies, Mona Kingston Jamaica; ^3^ Global Blood Therapeutics South San Francisco California; ^4^ Department of Hematology, Amsterdam University Medical Centers University of Amsterdam Amsterdam The Netherlands

To the Editor:

Leg ulcers are a common, painful, and debilitating chronic complication of sickle cell disease (SCD).^1^ The prevalence of this complication varies by geography, genotype, and age.^1^ In the United States, 14% to 18% of patients with SCD may develop leg ulcers at some point, and an estimated 1% to 5% of patients with SCD have active ulcers.^1^, ^2^, ^3^, ^4^ The incidence of leg ulcers in SCD remains steady despite advances in preventive strategies and supportive care.^2^ Patients typically develop their first leg ulcer in the second decade of life, and are at an increased risk for leg ulcers with increasing age and severity of anemia.^1^, ^5^ However, the true incidence of leg ulcers in SCD may be underestimated given the paucity of registries and large prospective trials for this complication.^1^

Leg ulcers in SCD tend to develop in areas with poor blood flow and limited subcutaneous tissue, such as the medial and lateral malleoli (ankles), and when untreated, can progress from epidermal skin loss to extensive tissue destruction and necrosis.^6^ The pain that results from ulcers is excruciating and markedly different from the pain associated with sickle cell crises.^1^, ^7^ Sharp pain often precedes the formation of new ulcers, and the severity of pain is often unrelated to the size of the formed ulcer.^1^ The intense, irregular pain, associated gait abnormalities, and lower limb deformities result in substantial physical and emotional impairment in these patients.^6^, ^7^

No standardized protocol for the care of these wounds exists.^8^ While nearly all patients with SCD have anemia, those with more severe hemolytic anemia are more prone to developing leg ulcers.^9^ Leg ulcers are almost exclusively found in SCD patients with a severe genotype (HbSS/HbSβ^0^) and are rarely found in milder forms of SCD (HbSC/HbSβ^+^).^10^ The SCD patients with a severe genotype are also at risk of developing other hemolysis‐related complications, including stroke, pulmonary hypertension, and renal dysfunction.^9^ While hydroxyurea and chronic transfusions are available for the treatment of SCD, there are no clinical data from systematic or controlled trials demonstrating their efficacy in the treatment of leg ulcers.^1^ Hydroxyurea has also been associated with ulcer formation in other hematological disorders and in SCD, based on several case reports.^1^

Voxelotor is a sickle hemoglobin (HbS)‐polymerization inhibitor approved for the treatment of SCD in patients aged ≥12 years. In the phase 3, randomized, double‐blinded, placebo‐controlled, HOPE trial (NCT03036813), treatment with voxelotor 1500 and 900 mg daily demonstrated rapid and sustained improvements in hemoglobin (Hb) levels and hemolysis markers compared with placebo. Here, we report on a post hoc analysis that evaluated the incidence of leg ulcers and their outcomes in patients enrolled in the HOPE trial across the 72‐week treatment period.

In the HOPE study, the presence of SCD‐related leg ulcers was assessed every 2 weeks for the first 8 weeks, every 4 weeks up to week 24, and every 12 weeks up to week 72. Using a dedicated leg ulcer electronic case report form at each time point, ulcer severity was graded as mild, moderate, or severe. Mild severity was defined as asymptomatic. Moderate severity was defined as an adverse event needing minimal, local, or noninvasive intervention. Severe was defined as an adverse event that was medically significant but not life‐threatening.

Among all enrolled patients, 33 of 274 (12.0%) reported having a history of leg ulcers at screening, corroborating previously reported incidences of SCD‐related leg ulcers. At study initiation, 13 of 274 (4.7%) patients had active leg ulcers (Table S1). Among these patients, four were in the voxelotor 1500 mg group (two with ulcers of mild severity, two with ulcers of moderate severity), six were in the voxelotor 900 mg group (three mild, three moderate), and three were in the placebo group (all mild). Baseline characteristics for these patients were generally similar between treatment groups. During the 72‐week treatment period, nine additional patients developed new ulcers (Figure 1(A)), for a total incidence of active leg ulcers of 22 of 274 (8.0%) patients in the HOPE study population. Of these nine patients, one was in the voxelotor 1500 mg group (mild severity), three were in the voxelotor 900 mg group (two mild, one moderate), and five were in the placebo group (three mild, two moderate). Only one patient who developed new ulcers during the study reported having a history of leg ulcers at screening (placebo group, moderate severity).

**FIGURE 1:**
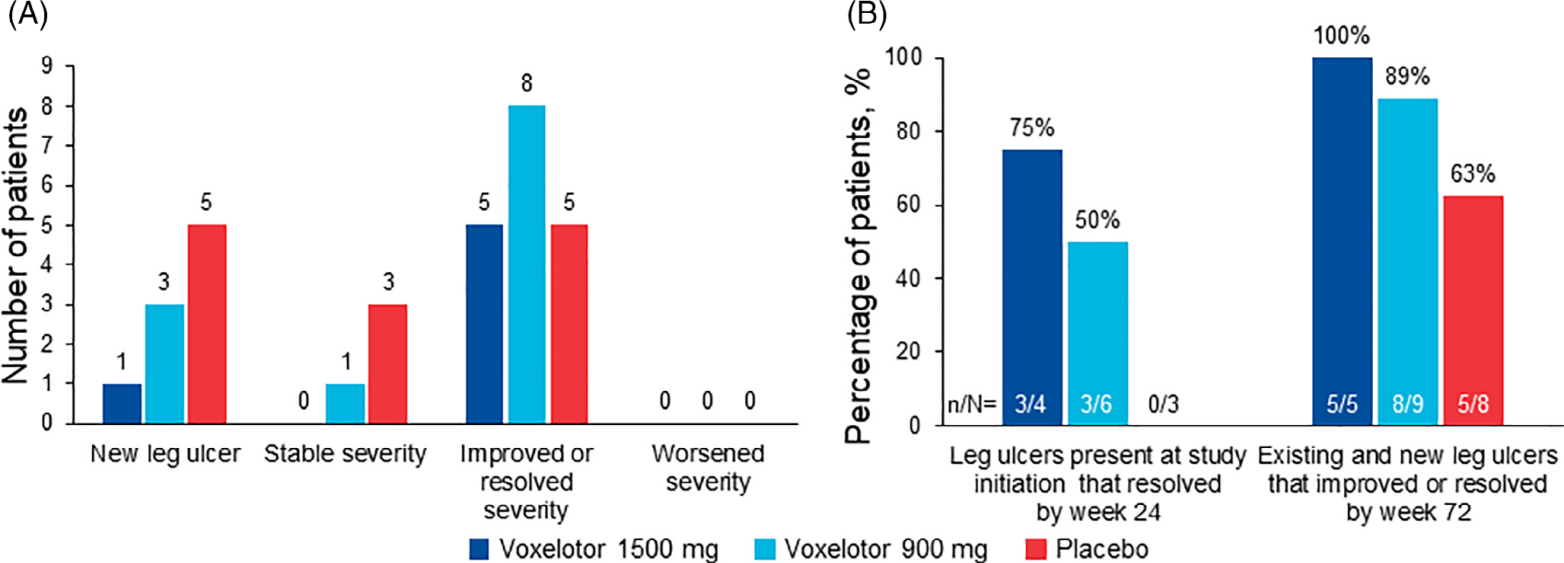
Change in leg ulcer severity across treatment groups during the 72‐week treatment period. (A) Numbers of patients who developed new leg ulcers, showed stable ulcer severity, showed improved or resolved ulcer severity, and showed worsened ulcer severity during the 72‐week treatment period. (B) Percentage of patients with leg ulcers present at study initiation that resolved by week 24 and percentage of patients with existing and new leg ulcers that improved or resolved by week 72

Overall, five of five (100%) patients in the voxelotor 1500 mg group, eight of nine (89%) in the 900 mg group, and five of eight (63%) in the placebo group had their leg ulcers improve or resolve by week 72 (Figure 1(B)). Among those with leg ulcers at initiation, three of four (75%) receiving voxelotor 1500 mg and three of six (50%) receiving voxelotor 900 mg had their leg ulcers resolve within 24 weeks, compared with none (zero of three) in the placebo group. For patients who developed new leg ulcers during the study, one of one in the voxelotor 1500 mg group, three of three in the voxelotor 900 mg group, and three of five in the placebo group had their ulcers resolve within 24 weeks.

The resolution of leg ulcers was associated with increased Hb levels. Among patients receiving voxelotor (1500 and 900 mg) who had leg ulcers at study initiation, leg ulcers resolved within 24 weeks for five of seven (71.4%) patients who achieved a Hb increase of >1.0 g/dL by week 24 (voxelotor 1500 mg, two patients; voxelotor 900 mg, three patients) compared with one of three (33.3%) patients who did not achieve a Hb increase of >1.0 g/dL by week 24. Among patients with leg ulcers at baseline, treatment with voxelotor was also associated with greater reductions in markers of hemolysis than placebo (Table S2). Resolution of leg ulcers by week 24 was associated with higher percentages of Hb occupancy, defined as percentage of Hb‐voxelotor binding (voxelotor 1500 mg [n = 3], 28.1%; voxelotor 900 mg [n = 3], 15.5%), and greater increases in Hb levels (voxelotor 1500 mg [n = 3], 1.5 g/dL; voxelotor 900 mg [n = 3], 3.0 g/dL) compared with those of patients whose ulcers did not resolve (Table S3). No new safety signals were observed among patients with leg ulcers, and treatment with voxelotor was well tolerated.

In this post hoc analysis, nearly all patients (>90%) receiving voxelotor (1500 and 900 mg doses) had their leg ulcers improve or resolve by week 72. Improvements in Hb levels and reduction in markers of hemolysis associated with voxelotor treatment in the current analysis were similar to those observed in the larger HOPE study.^11^ Most patients (>70%) receiving voxelotor had their ulcers resolve within 24 weeks. The resolution of ulcers was generally associated with concordant increases in Hb, decreases in hemolysis markers, and greater Hb occupancy at 24 weeks.

While the pathophysiology of ulcer formation in SCD remains incompletely understood, it is likely caused by a combination of systemic and local factors, including chronic inflammation, hemolysis, and venostasis.^1^ Patients with leg ulcers have also been noted to have impaired red blood cell deformability and altered rheology that was associated with increased intravascular hemolysis.^1^ Patients with SCD presenting with leg ulcers should be screened for end‐organ complications, given the risk for other forms of organ damage.^9^

As a HbS‐polymerization inhibitor, voxelotor has been demonstrated to improve parameters of red blood cell health and attenuate intravascular hemolysis in patients.^11^ In the current study, patients with a Hb increase of >1.0 g/dL with voxelotor and a higher occupancy rate were most likely to have their leg ulcers resolve. A limitation of this study is the small sample size of participants with leg ulcers.

Leg ulcers are a debilitating, painful, and difficult‐to‐treat complication that is common in patients with SCD. Most available treatments for leg ulcers with SCD do not focus on treating the underlying pathophysiology of the condition. The results of this study suggest that voxelotor presents a potential clinical benefit for patients with SCD and leg ulcers and support future study in a prospective clinical trial.

## CONFLICT OF INTEREST

Caterina P. Minniti: Global Blood Therapeutics, consultant, HOPE trial investigator; Jennifer Knight‐Madden: Global Blood Therapeutics, HOPE trial investigator; Bart J. Biemond: Global Blood Therapeutics, advisory boards, HOPE trial investigator; Margaret Tonda, Sarah Gray: Global Blood Therapeutics, employees, stockholder; Joshua Lehrer‐Graiwer: Global Blood Therapeutics, former employee, stockholder.

## ETHICS STATEMENT

This trial was conducted in accordance with the principles of the Declaration of Helsinki International Conference on Harmonization, Good Clinical Practice guidelines in clinical trials, and all applicable country‐specific regulatory guidelines. The study protocol was reviewed and approved by institutional review boards and independent ethics committee of each of the participating trial sites.

## PATIENT CONSENT STATEMENT

Written informed consent was obtained from patients prior to study.

## Supporting information


**Table S1**. Patient characteristics and demographics among patients with leg ulcers at initiation

**Table S2**. Change from baseline in hematologic parameters at week 24 in patients with leg ulcers at study initiation

**Table S3**. Change from baseline in hematologic parameters at week 24 in patients with leg ulcers at study initiation that resolved by week 24

## Data Availability

The data that support the findings of this study are available on request from the corresponding author. The data are not publicly available due to privacy or ethical restrictions.
