# Supplementary material for: The impact of voxelotor treatment on leg ulcers in patients with sickle cell disease
Source: Am J Hematol. 2021 Feb 19;96(4):E126–8. doi: 10.1002/ajh.26101 (PMC7986764; doi:10.1002/ajh.26101)
Supplement: Supplementary file 1 — Table S1. Patient characteristics and demographics among patients with leg ulcers at initiation [file AJH-96-E126-s001.docx]

**Table S1. Patient characteristics and demographics among patients with leg ulcers at initiation**

|  | Voxelotor  1500 mg  (n=4) | Voxelotor  900 mg  (n=6) | Placebo  (n=3) |
| --- | --- | --- | --- |
| Age, median (range), years | 23.5 (12-52) | 21 (20-35) | 42 (34-45) |
| Male, % | 50 | 83 | 67 |
| HbSS genotype, % | 75^a^ | 100 | 100 |
| Baseline indirect bilirubin, mean, μmol/L | 34 | 64 | 51 |
| Baseline lactate dehydrogenase, mean, U/L | 541 | 431 | 787 |
| Baseline absolute reticulocytes, mean, ×10^9^/L | 369 | 378 | 308 |
| Baseline Hb, mean, g/dL | 8.1 | 7.9 | 7.3 |
| Severity of leg ulcers at initiation, n  Severe  Moderate  Mild | 0  2  2 | 0  3  3 | 0  0  3 |

^a^One patient in the voxelotor 1500 mg group had sickle cell variant (C.20A>T, Glu6Val) genotype.
Hb, hemoglobin; HbSS, homozygous for SCD.
