# Supplementary material for: The impact of voxelotor treatment on leg ulcers in patients with sickle cell disease
Source: Am J Hematol. 2021 Feb 19;96(4):E126–8. doi: 10.1002/ajh.26101 (PMC7986764; doi:10.1002/ajh.26101)
Supplement: Supplementary file 2 — Table S2. Change from baseline in hematologic parameters at week 24 in patients with leg ulcers at study initiation [file AJH-96-E126-s003.docx]

**Table S2. Change from baseline in hematologic parameters at week 24 in patients with leg ulcers at study initiation**

|  | Voxelotor  1500 mg  (n=4) | Voxelotor  900 mg  (n=6) | Placebo  (n=3) |
| --- | --- | --- | --- |
| Hb occupancy, mean, % | 24.2 | 14.9 | - |
| Indirect bilirubin percent change from baseline, mean, % | –8.2 | –49.6 | 9.0 |
| Lactate dehydrogenase percent change from baseline, mean, % | –3.2 | –19.7 | –17.2 |
| Absolute reticulocytes percent change from baseline, mean, % | –3.2 | –28.2 | 49.2 |
| Hb change from baseline, mean, g/dL | 1.1 | 2.4 | 0.2 |

Hb, hemoglobin.
