# Supplementary material for: The impact of voxelotor treatment on leg ulcers in patients with sickle cell disease
Source: Am J Hematol. 2021 Feb 19;96(4):E126–8. doi: 10.1002/ajh.26101 (PMC7986764; doi:10.1002/ajh.26101)
Supplement: Supplementary file 3 — Table S3. Change from baseline in hematologic parameters at week 24 in patients with leg ulcers at study initiation that resolved by week 24 [file AJH-96-E126-s002.docx]

**Table S3. Change from baseline in hematologic parameters at week 24 in patients with leg ulcers at study initiation that resolved by week 24**

|  | Voxelotor  1500 mg  (n=3) | Voxelotor  900 mg  (n=3) |
| --- | --- | --- |
| Hb occupancy, mean, % | 28.1 | 15.5 |
| Indirect bilirubin percent change from baseline, mean, % | –10.3 | –65.4 |
| Lactate dehydrogenase percent change from baseline, mean, % | 2.4 | –26.6 |
| Absolute reticulocytes percent change from baseline, mean, % | –10.7 | –17.0 |
| Hb change from baseline, mean, g/dL | 1.5 | 3.0 |

Hb, hemoglobin.
